# The Effect of Geography and Citizen Behavior on Motor Vehicle Deaths in the United States

**DOI:** 10.1371/journal.pone.0123339

**Published:** 2015-04-07

**Authors:** Nicole Abaid, James Macinko, Diana Silver, Maurizio Porfiri

**Affiliations:** 1 Department of Biomedical Engineering and Mechanics, Virginia Polytechnic Institute and State University, Blacksburg, Virginia, United States of America; 2 Departments of Health Policy and Management and Community Health Sciences, University of California Los Angeles, Los Angeles, California, United States of America; 3 Department of Nutrition, Food Studies, and Public Health, New York University, New York, New York, United States of America; 4 Department of Mechanical and Aerospace Engineering, New York University Polytechnic School of Engineering, Brooklyn, New York, United States of America; Johns Hopkins Bloomberg School of Public Health, UNITED STATES

## Abstract

Death due to motor vehicle collisions (MVCs) remains a leading cause of death in the US and alcohol plays a prominent role in a large proportion of these fatalities nationwide. Rates for these incidents vary widely among states and over time. Here, we explore the extent to which driving volume, alcohol consumption, legislation, political ideology, and geographical factors influence MVC deaths across states and time. We specify structural equation models for extracting associations between the factors and outcomes for MVC deaths and compute correlation functions of states’ relative geographic and political positions to elucidate the relative contribution of these factors. We find evidence that state-level variation in MVC deaths is associated with time-varying driving volume, alcohol consumption, and legislation. These relationships are modulated by state spatial proximity, whereby neighboring states are found to share similar MVC death rates over the thirty-year observation period. These results support the hypothesis that neighboring states exhibit similar risk and protective characteristics, despite differences in political ideology.

## Introduction

Human interactions within a society create complex systems, which depend on phenomena at both individual and group levels [1]. Such systems are influenced by a variety of formal and informal rules, which reflect desirable outcomes for the society and behaviors to achieve them [2]. In this context, public policies are powerful tools, yet many aspects of their adoption and diffusion are not well understood [3].

Over the past three decades, states in the US have expanded the use of public policies, such as laws, regulations, taxes, and other enforcement mechanisms, to attain public health goals and to facilitate the change of complex health behaviors [4–7]. In spite of growing evidence for the effectiveness of numerous individual laws, important public policy questions remain unanswered, such as how different combinations of health laws and policies work in concert or at odds with one another, how states’ internal political and economic profiles affect the policy-making process, and whether states that are geographically or politically similar tend to emulate one another with regard to policies intended to tackle public health problems.

The study of complex systems [8, 9] offers novel approaches for handling large datasets. For example, public health problems (e.g., tobacco control and pandemic flu [10–14]), the spread of invasive species driven by the global shipping industry [15], models of energy flows evidencing climate [16], and analysis of cholera epidemics [17] have already benefitted from these approaches. Complex systems tools may be particularly beneficial for the analysis of policymaking among states, as they allow for a quantitative understanding of the complex interactions within a group of interconnected units (e.g., policymakers) who are responsible for the production of an observable and emergent phenomenon (e.g., state health policy landscape) [18], for example to study the relationship between adopting mandatory seat belt laws and preventing injury and death as a result of traffic accidents [19].

A pressing public health problem is to understand and predict the relationship between alcohol consumption, motor vehicle collisions (MVCs), and traffic fatalities. Although traffic fatality rates have been halved during the past thirty years, MVCs remain the second largest contributor to years of life lost before age 75 in the US and are the single largest cause of death among youth aged fifteen to twenty-four [20, 21]. Moreover, MVC-related death rates vary widely among states, from 0.84 deaths per 100 million vehicle miles of travel in Iowa to 4.35 deaths per 100 million vehicle miles of travel in New Jersey.

Alcohol use is a major contributor to MVCs and was involved in nearly one-third of all fatal crashes in 2009 [22]. Much of the decline in deaths over the past thirty years has been attributed to more effective state policies regulating motor vehicle safety and reducing drunk driving. Yet, despite early progress, the rate of alcohol involvement in fatal collisions has remained near 32% since 1994 and currently ranges from about 25% in Kentucky to 48% in Hawaii [22]. In this context, we posit that complex systems approaches may offer fundamental insight into temporal and spatial processes underlying the persistence of this public health problem.

In this paper, we explore the dynamics of state MVC deaths and the factors associated with changes in death rates over time. We investigate the extent to which a set of key state characteristics and geographical factors influence MVC deaths. MVC deaths are measured by total counts of deaths, rates of deaths per vehicle miles travelled, and the percentage of deaths that are alcohol-related. Explanatory variables comprise the driving volume (the total amount of vehicle miles travelled in each state and year), alcohol consumption (gallons per capita), legislation (the proportion of an ensemble of alcohol- and driving-related laws adopted), and political ideology. The relationships between MVC deaths and explanatory variables are studied using structural equation models (SEMs) on data from all fifty states over a recent thirty year period. SEMs are selected as they enable the simultaneous measurement of relationships among multiple independent variables and a dependent variable, which allows for assessing potential confounders between the variables. Spatial and political measures are leveraged to determine how state geographic and ideological similarities contribute to such relationships. Based on the knowledge that the interactions among states are able to inform their public policies, we expect to find significant dependencies between MVC deaths and the selected variables. We further anticipate that states that are geographically closer and ideologically similar exhibit comparable patterns.

## Results

### MVC outcomes and relationship to alcohol

**Fig 1:**
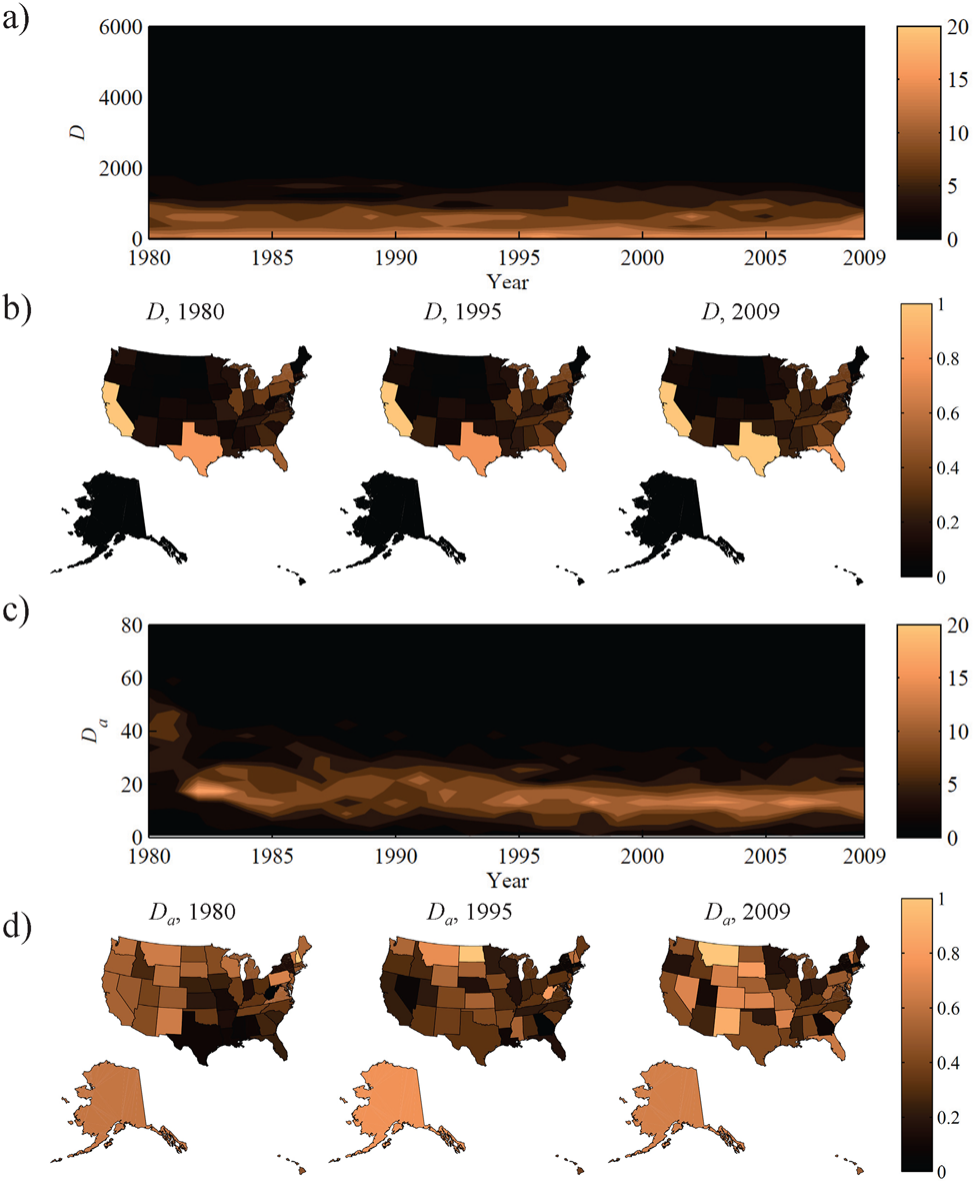
Synopsis of MVC deaths and the percentage of which are related to alcohol. a) Contour plot of the frequency distribution of MVC deaths (raw counts) *D* over all states computed annually for 30 years. Dark/light colors indicate small/large numbers of states with the given value of *D*. b) Snapshots of *D* normalized between zero and one for three representative years. c) Contour plot of the frequency distribution of the percentage of MVC deaths related to alcohol *D_a_* for all states over thirty years. Dark/light colors indicate small/large numbers of states with the given value of *D_a_*. d) Snapshots of *D_a_* values normalized between zero and one for three representative years. All colored maps were created with the MATLAB Mapping toolbox.

We find that MVC deaths, denoted *D*, have decreased at the national level from 51,050 in 1980 to 32,788 in 2010. Most states experienced a decline over time, but overall rates varied largely by state, see Fig 1a. This variation is further demonstrated in Fig 1b, which shows *D* at three evenly spaced years in the studied thirty year period, 1980, 1995, and 2009. In this figure, *D* is normalized over all states with respect to the annual maximum value; thus, the state with largest *D* is shown with a value of 1 and the state with the smallest *D* is shown with a value of 0 for each map, where numerical values are given in the color bar. We consistently see that the large and populous states- CA, FL, and TX- have the highest number of MVC deaths. Interestingly, although NY is both large and populous, it fails to reach similarly high values of *D*.

The percentage of MVC deaths involving alcohol, denoted *D*_*a*_, shows similar nationwide trends to *D*, decreasing from 37.0% in 1980 to 18.3% in 2010. The variation of this quantity between states is generally narrow, see Fig 1c. However, considering *D*_*a*_ normalized over all states in 1980, 1995, and 2009, we see that the footprint of alcohol-related traffic deaths is more complex than the previous case, see Fig 1d. The patterns among the states do not appear to be constant over time, nor are they dependent on geographic location over time.

### Association between MVC outcomes and potential determinants of variation among states

**Fig 2:**
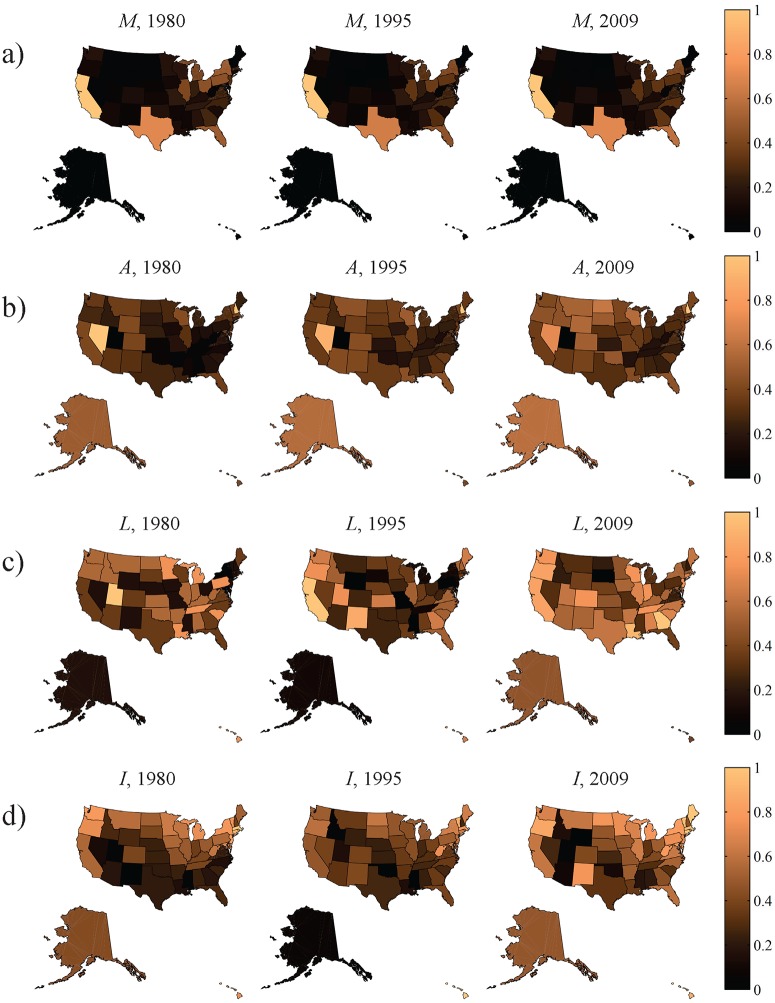
Snapshots of state characteristics for three representative years. Characteristics are: a) vehicle miles travelled *M*, b) per capita alcohol consumption *A*, c) proportion of relevant laws adopted *L*, and d) state political ideology *I*. Each characteristic is normalized between zero and one for all states independently for each year. All colored maps were created with the MATLAB Mapping toolbox.

**Fig 3:**
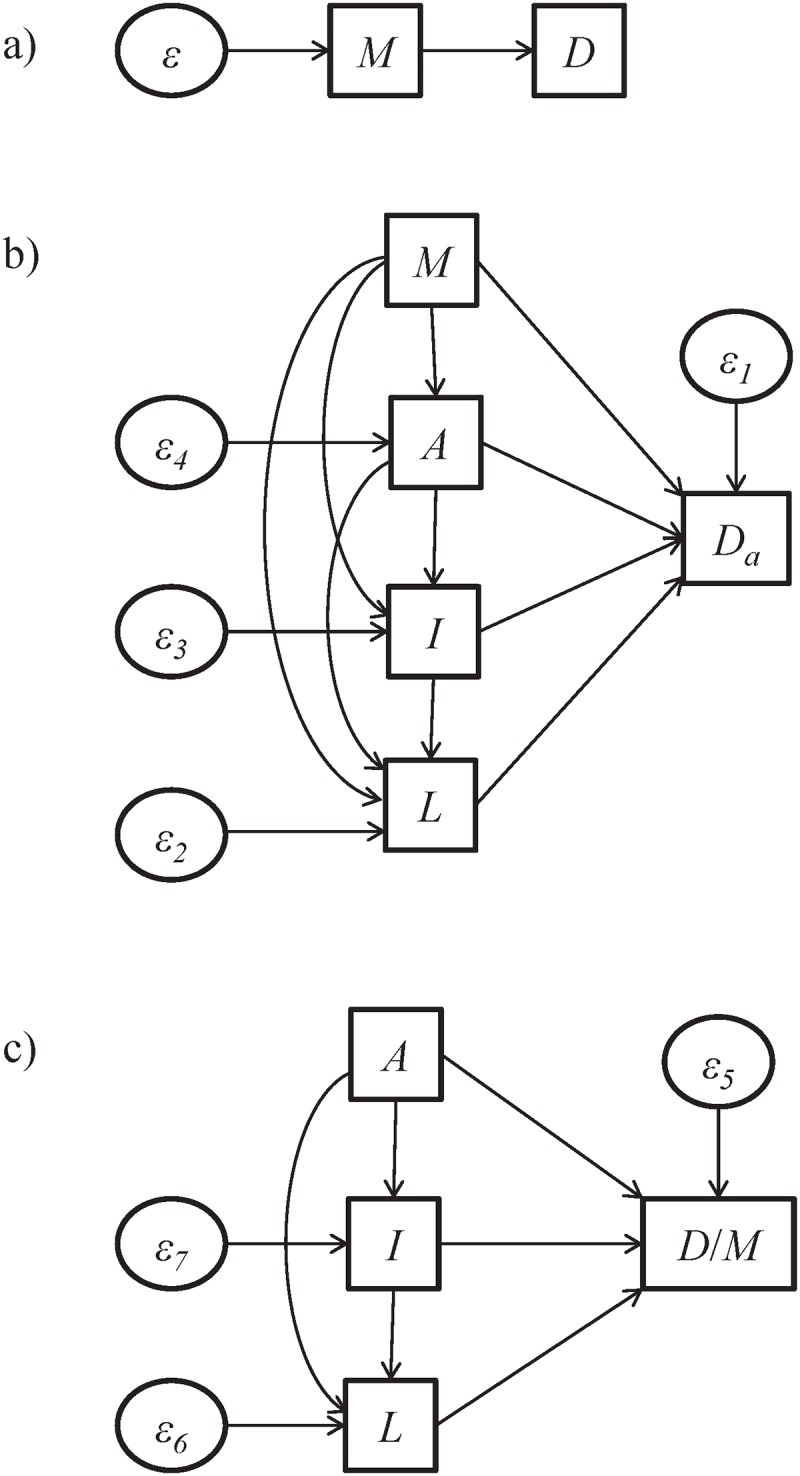
Path diagram of structural equation models for state characteristics and outcomes. Each variable is normalized between 0 and 1 by dividing all values by its largest value in the thirty year time span. SEM diagrams are shown for the models in a) Eq (1), b) Eq (2), and c) Eq (3). All relationships, shown as arrows, denote free parameters identified by the model and given in Table 1 with their statistical significances.

We analyze four state descriptors: vehicle miles travelled (*M*), per capita alcohol consumption (*A*), the proportion of selected laws related to reducing risky and drunk driving and increasing driver and occupant protection (e.g. seatbelts) (*L*), and state ideology (*I*). The state ideology is between 0 and 1, with 1 indicating most liberal political ideology. The computation of these descriptors is described in detail in the Materials and Methods section. These quantities are reported nationwide, normalized with respect to the annual maximum values over all states, in 1980, 1995, and 2009 in Fig 2. The results from the SEMs are presented in Table 1 and summarized in network form in Fig 3.

**Table 1:** Results of structural equation models.

All years
*D*_*i*_ *= 0*.93*M_*i*_ + 0.022* + 0.005*∈_i_
*D*_*ai*_ = -0.18*M_*i*_ + 0.32*A_*i*_—0.19*L_*i*_—0.043*I*_*i*_ + 0.27* + 0.013*∈_1i_
*L*_*i*_ = 0.24*M_*i*_—0.45*A_*i*_ + 0.47*I_*i*_ + 0.20* + 0.033*∈_*2i*_
*I*_*i*_ = 0.032**M_*i*_ + 0.073*A_*i*_ + 0.72* + 0.007*∈_*3i*_
*A*_*i*_ = -0.11*M_*i*_ + 0.43* + 0.010*∈_*4i*_
(*D/M*)_*i*_ = 0.13*A_*i*_—0.37*L_*i*_—0.57*I_*i*_ + 0.88* + 0.009*∈_*5i*_
*L*_*i*_ = -0.51*A_*i*_ + 0.49*I*_*i*_ + 0.24* + 0.034*∈_*6i*_
*I*_*i*_ = 0.065*A_*i*_ + 0.73* + 0.007*∈_*7i*_
**1980s**
*D*_*i*_ = 1.49*M_*i*_ + 0.007** + 0.002*∈_*i*_
*D*_*ai*_ = -0.27*M_*i*_ + 0.39*A_*i*_—0.25*L_*i*_—0.057*I*_*i*_ + 0.27* + 0.019*∈_*1i*_
*L*_*i*_ = -0.007*M*_*i*_—0.27*A_*i*_ + 0.12**I_*i*_ + 0.25* + 0.012*∈_*2i*_
*I*_*i*_ = 0.033*M*_*i*_ + 0.057*A*_*i*_ + 0.72* + 0.007*∈_*3i*_
*A*_*i*_ = -0.061*M*_*i*_ + 0.46* + 0.014*∈_*4i*_
(*D/M*)_*i*_ = 0.11**A_*i*_—0.23*L_*i*_—0.78*I_*i*_ + 1.07* + 0.012*∈_*5i*_
*L*_*i*_ = -0.27*A_*i*_ + 0.12**I_*i*_ + 0.25* + 0.012*∈_*6i*_
*I*_*i*_ = 0.055*A*_*i*_ + 0.72* + 0.007*∈_*7i*_
**1990s**
*D*_*i*_ = 0.95*M_*i*_ + 0.012* + 0.002*∈_*i*_
*D*_*ai*_ = -0.24*M_*i*_ + 0.18*A_*i*_—0.16*L_*i*_—0.10*I*_*i*_ + 0.35* + 0.011*∈_*1i*_
*L*_*i*_ = 0.089*M_*i*_—0.023*A*_*i*_ + 0.021*I*_*i*_ + 0.37* + 0.011*∈_*2i*_
*I*_*i*_ = 0.012*M*_*i*_ + 0.057*A*_*i*_ + 0.73* + 0.005*∈_*3i*_
*A*_*i*_ = -0.078*M_*i*_ + 0.40* + 0.007*∈_*4i*_
(*D/M*)_*i*_ = 0.020*A*_*i*_—0.13*L_*i*_—0.54*I_*i*_ + 0.77* + 0.005*∈_*5i*_
*L*_*i*_ = -0.044*A*_*i*_ + 0.026*I*_*i*_ + 0.39* + 0.011*∈_*6i*_
*I*_*i*_ = 0.054*A*_*i*_ + 0.73* + 0.005*∈_*7i*_
**2000s**
*D*_*i*_ = 0.79*M_*i*_ + 0.009* + 0.002*∈_*i*_
*D*_*ai*_ = -0.12*M_*i*_ + 0.24*A_*i*_—0.17*L_*i*_ + 0.012*I*_*i*_ + 0.23* + 0.009*∈_*1i*_
*L*_*i*_ = 0.15*M_*i*_—0.18**A_*i*_ + 0.40*I_*i*_ + 0.32* + 0.018*∈_*2i*_
*I*_*i*_ = 0.024*M*_*i*_ + 0.20*A_*i*_ + 0.69* + 0.007*∈_*3i*_
*A*_*i*_ = -0.091*M_*i*_ + 0.42* + 0.006*∈_*4i*_
(*D/M*)_*i*_ = -0.003*A*_*i*_—0.087*L_*i*_—0.42*I_*i*_ + 0.64* + 0.004*∈_*5i*_
*L*_*i*_ = -0.25*A_*i*_ + 0.42*I_*i*_ + 0.36* + 0.019*∈_*6i*_
*I*_*i*_ = 0.19*A_*i*_ + 0.70* + 0.007*∈_*7i*_

Coefficients with statistical significance p<0.01 and p<0.05 are denoted by * and **, respectively.

Comparing the dependence of *D* on *M*, we find significant positive associations when the states are partitioned into decades and when they are combined over the thirty years. From this analysis, we notice that the regression coefficients are positive and decrease in time. Nevertheless, such analysis using the raw number of MVC deaths does not allow for an objective comparison between states of varying populations and geographic sizes. Thus, we consider *D*/*M* as the focal outcome in the rest of the analyses, which eliminates the over-emphasis of states with higher populations or more roads.

Studying the dependence of *D*_*a*_ on the state characteristics, we find a significant association with *M* over all decades and years and the regression coefficients change through the decades; these negative coefficients increase towards zero over time. The association with *A* shows significant positive associations over all decades and all years combined. Here, the regression coefficients vary non-monotonically over the decades. Significant negative associations are found between *L* and *D*_*a*_ when the decades are considered separately and when all years are combined. The regression coefficients show a strong increase from the 1980s to the 1990s, after which they stay approximately constant. Considering *D*_*a*_’s variation with *I*, we do not find significant association for any decade, nor when combining all years.

Considering the association between *D/M* and *A*, *L*, and *I*, we find a significant and positive association with *A* for all years combined and in the 1980s separately; for the 1990s and the 2000s, we do not find any association for the regression coefficients. We further find significant negative associations between *L* and *I* when the decades are considered separately and when all years are combined. The regression coefficients are negative and increase toward zero with time, by an approximate factor of 1.5 per decade.

Among the independent factors in these two SEMs, we find a complex pattern of dependence among factors and over time. Notably, we see that *L* has significant positive and negative associations with *M* and *A*, respectively, over all years and in all but one decade in the SEM with *D*_*a*_. The relationship between *A* and *L* is further mirrored in the SEM with *D*/*M*. For the computed errors, we find that the coefficients mitigating possible time-varying confounding factors are relatively small compared to the coefficients between pairs of factors.

### Similarity analysis of state pairs

**Fig 4:**
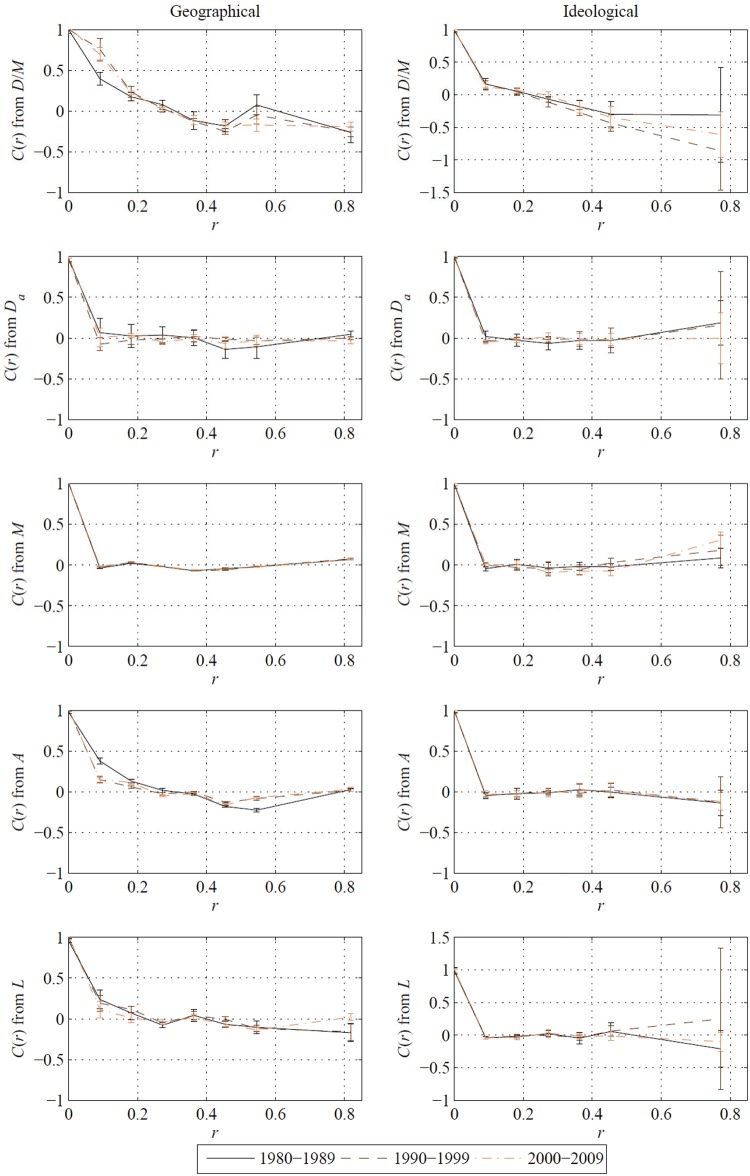
Mean correlation function for state characteristics and outcomes, computed using geographic and ideological distance as indicated by the column heading. Horizontal axes show distance *r* and vertical axes *C*(*r*). Distances are normalized from zero to one based on the maximum distance between state centers and discretized into eleven equal bins. The largest four bins are combined to lessen the effect of sparse occupancy at large distances, and the value for the correlation function of the set of state pairs in the combined bin is shown at 0.82 on the horizontal axis. Points indicate mean value and error bars one standard deviation taken over selected decade.

Following the analysis in Gallos et al. [23], we compute a correlation function to identify trends between different state characteristics and the MVC outcomes for pairs of states separated by a certain “distance”. The distance between states is defined in two ways: the location of geographic state center and the political ideology of the state population. A correlation coefficient is evaluated for all pairs of states separated by a given distance, computed in any of the previously defined outcomes and characteristics. Further technical details are provided in the Materials and Methods section. Fig 4 shows the correlation function computed using geographic and ideological distances versus *D/M*, *D*_*a*_, *M*, *A*, and *L*.

Considering the left column of Fig 4, we find that the outcome *D/M* and characteristics *A* and *L* show positive correlations for small geographic distances, indicating that nearby states are more similar than states that are further apart. These short-distance correlations vary in time, whereby *D/M* tends to be more correlated as time increases (greater than one standard deviation difference in the mean values) and *A* displays an opposite trend. Correlations of the state characteristics and outcomes generally decrease with increasing geographic distances, converging near zero. In a few cases, such correlations tend to negative values, as for *D/M* which attains negative values for geographic distances larger than 0.4.

**Fig 5:**
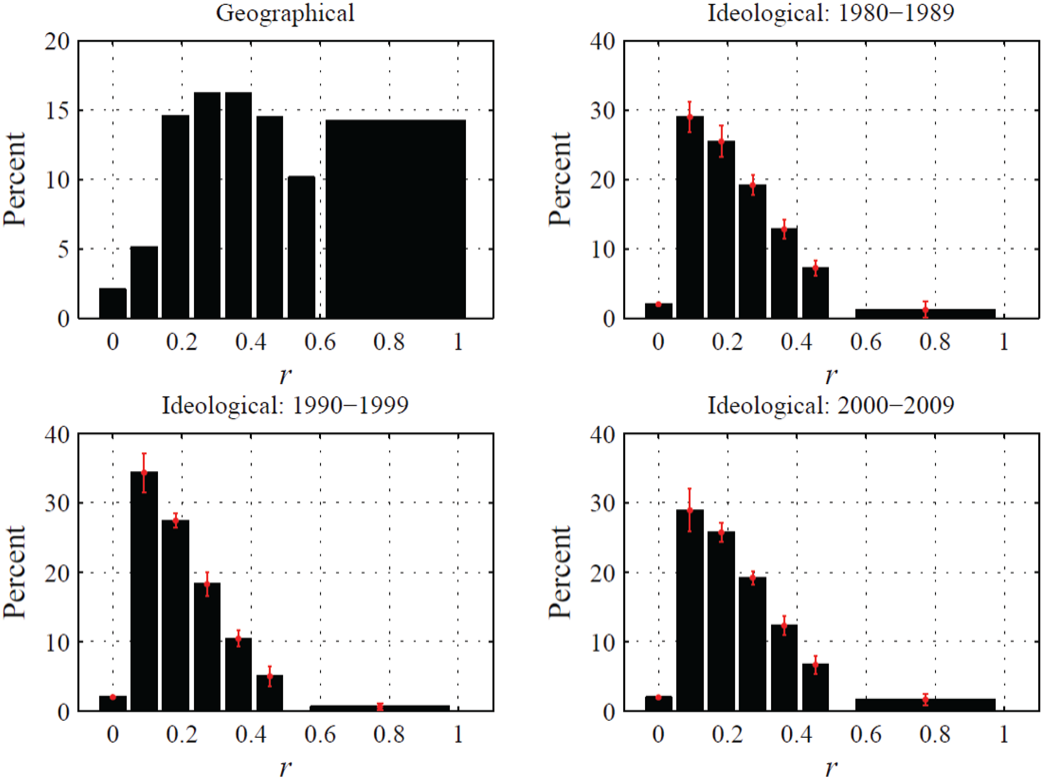
Histograms of frequency of state pairs separated by a distance *r* computed using geographic location and political ideology. Horizontal axes show distance and vertical axes percentage of state pairs. Distances are normalized from zero to one and discretized into eleven equal bins; the largest four are combined to lessen the effect of sparse occupancy at large distances. Types of distances are given in the subfigure titles. For subfigures using ideological distances, error bars indicate one standard error over the selected decade.

When we use ideological differences to define distances, we uncover strikingly different patterns than in the previous case, see the right column of Fig 4. For no quantity except *D/M* do we find any correlation among near neighbors, whereby states which are ideologically similar display correlations near zero. Increasing the ideological distance does not produce noticeable variations, except for *D/M* which attains nonnegligible negative values for distances larger than 0.3. In contrast to our analysis of geographic distances, the data variation remarkably increases with growing distance. Such wide variations are likely due to the smaller number of state pairs which are ideologically very dissimilar, see Fig 5.

## Discussion

This study supports the hypothesis that MVC deaths are associated with a set of policy and environmental factors, which explains a large proportion of state variations in outcomes. Moreover, these factors (i.e., *M*, *A*, and *L*) are modified by state characteristics and are influenced by geographic proximity and less so by ideological differences, as demonstrated through the SEM results.

Our results confirm the notion that the main measure of risk of an MVC is the amount of time spent driving and suggest that, in spite of increased driving volume, the risk of MVC deaths has decreased over the past three decades. One explanation for this trend has been the increased attention towards the design of safer vehicles [24]. However, our findings support the importance of regulations promoting safer driving behavior in reducing MVC deaths. Specifically, we find that MVC death rate and the proportion of those related to alcohol are negatively associated with the number of laws aimed at reducing drunk driving and promoting driver and passenger safety. Similarly, we observe that MVC deaths per miles travelled decreases as the state political ideology grows more liberal, although liberal states do not necessarily have a larger number of laws.

We also confirm that the percentage of alcohol-related MVC deaths increases with per capita alcohol consumption [25] in line with intuitive expectations. Interestingly, we further observe that the alcohol-related MVC deaths are negatively associated with driving volume, whereby in states where the amount of driving is higher, the percentage of alcohol-related MVC deaths is lower. This evidence is unlikely to be explained by variations in the legislation across states, since we find no association between the driving volume and the number of laws.

Considering results from the geographic analysis, we find that states’ physical proximity is a predictor of MVC deaths, whereby states that are close by tend to have similar MVC deaths per unit vehicle mile. Likely, this reflects the composition of the transportation infrastructure in the US, where high density urban centers are connected through interstate highways and smaller shared roads, while low traffic volume areas are connected to similar areas. However, the role of physical proximity appears to be secondary in explaining state differences in alcohol-related deaths. The negative correlation between alcohol-related MVC deaths and traffic volume may be related to the lack of correlation we observe between traffic volume in physically proximal states. It may indicate that alcohol-related MVC deaths are associated with specific driving patterns involving smaller travel distances [26].

Another explanation for the importance of physical distances on MVC deaths is the finding that proximal states have similar legal environments. The political science literature suggests that, all else equal, states are more likely to adopt laws that have been successful in another state because this simplifies policymakers’ task of finding a solution to a given problem [27, 28]. There are several reasons why states should cooperate on laws regarding traffic fatalities. First, roads and highways cross state borders, necessitating communication and coordination among law enforcement agencies and state departments of transportation. Second, policymakers and policy analysts in neighboring states may have opportunities to learn from one another through regional associations and conferences or other formal mechanisms to exchange information. They may also be more willing to look to neighboring states for solutions to policy problems because of regional similarities in populations, geography, or economic circumstances. We note that state proximity also predicts similar patterns of alcohol consumption, reflecting an underlying risk factor for MVC deaths. This could also be a result of the fact that geographically contiguous states necessarily share some aspects of their legal environments, including alcohol, taxes, and sales restriction.

In contrast with our expectations, our results indicate that political ideology is less predictive of MVC deaths than geography and appears to exert an influence only for states which are ideologically very different. Specifically, states with different ideologies tend to be negatively correlated in their MVC deaths, suggesting that differences in political ideology may be associated with additional risk or protective factors for MVC deaths. One specific pathway identified through the SEM is that more liberal ideology is positively associated with greater laws intended to regulate behaviors which may explain its indirect effect on outcomes in different state contexts.

In conclusion, we have demonstrated that MVC deaths in US are related to multiple factors, spanning citizens’ and policy makers’ behaviors as well as inherent geographical and ideological factors determining state relationships. Moreover, influencing factors are somewhat different for MVC deaths overall compared to MVC deaths related to alcohol. This complexity hints at the need for stimulating further interdisciplinary research to elucidate the interplay between similarity and dissimilarity across states in shaping public health problems in the US.

## Materials and Methods

### Datasets

The study uses data from the State Health Policy Research Dataset (SHePRD), which includes traffic and alcohol policies from all 50 states 1980–2010 [29]. SHePRD is publicly available through ICPSR and includes data retrieved, validated, standardized, and merged from public use datasets, as well as original legal research. The data included in analysis of vehicle miles travelled, and state alcohol consumption are obtained from the NHTSA. MVC deaths per state come from the Fatal Accident Reporting System (FARS). MVC deaths are taken as total counts of annual deaths per state and the percentage of deaths that are alcohol-related. Total vehicle miles travelled is the statewide annual sum of the average amount of time motor vehicles are on the road multiplied by the length of roadway. This measure takes into account both the types of vehicles and the types of roadways on which they typically drive and it is officially computed for each state by the NHTSA. Alcohol consumption is the annual gallons of alcohol sold per capita. Laws were selected for inclusion in the dataset if there was evidence of their effectiveness in reducing morbidity and mortality in the literature. Binary variables in the dataset indicate the presence or absence of each law in each state and year during the study period and the proportion of these laws adopted (ranging from zero to one) is taken as an annual state descriptor.

State ideology measures the average location of the elected officials in each state on a liberal-conservative continuum based on weighted ideology scores for the governor and the major party delegations in each house of the state legislature derived from interest group ratings, opinion polls, and roll call data. The scale ranges from zero (representing the most conservative value) to one hundred (representing the most liberal position) and directly follows the procedure in [27]. However, this quantity is normalized between zero and one for the purposes of this study. We used the haversine formula to compute the distances between state geographic centers; Alaska and Hawaii were excluded from this calculation since they would result in artificially large distances. Statistics on the data used for the analysis are given in Table 2.

**Table 2:** Raw and normalized data used for statistical analysis.

	All years	1980s	1990s	2000s
***M***	4.7 x10^4^ ± 1.0x10^4^	3.5 x10^4^ ± 0.41 x10^4^	4.8 x10^4^ ± 0.38 x10^4^	5.8 x10^4^ ± 0.19 x10^4^
***M* (norm.)**	0.78 ± 0.17	0.58 ± 0.067	0.79 ± 0.063	0.96 ± 0.031
***A***	2.4 ± 0.19	2.6 ± 0.13	2.3 ± 0.087	2.4 ± 0.082
***A* (norm.)**	0.86 ± 0.067	0.94 ± 0.047	0.81 ± 0.031	0.84 ± 0.029
***L***	0.317 ± 0.134	0.170 ± 0.0656	0.312 ± 0.0357	0.470 ± 0.0504
***L* (norm.)**	0.59 ± 0.25	0.32 ± 0.12	0.59 ± 0.067	0.88 ± 0.095
***I***	0.759 ± 0.0265	0.748 ± 0.0232	0.751 ± 0.0158	0.776 ± 0.0306
***I* (norm.)**	0.93 ± 0.032	0.91 ± 0.028	0.92 ± 0.019	0.95 ± 0.037
***D*/*M***	2.00 ± 0.594	2.72 ± 0.412	1.81 ± 0.155	1.48 ± 0.128
***D*/*M* (norm.)**	0.57 ± 0.17	0.77 ± 0.12	0.51 ± 0.044	0.42 ± 0.036
***D***_***a***_	21.1 ± 5.19	25.9 ± 6.26	19.7 ± 1.97	17.6 ± 1.21
***D***_***a***_ **(norm.)**	0.55 ± 0.14	0.68 ± 0.16	0.51 ± 0.052	0.46 ± 0.032

For each variable, means are taken over the states and years and standard deviations are taken of the mean state values over the years. Results are shown as mean ± one standard deviation. We comment the normalized data is dimensionless and takes values between zero and one, while the raw data is dimensional following the definitions of the characteristics and outcomes.

### Analysis and statistics

We specify three structural equation models [30] to extract the associations between state characteristics and outcomes with p-values less than 0.01 and 0.05 used for significance; these models are shown in Eqs (1), (2), and (3) below. To ensure the feasibility of this analysis, and in particular that the covariance matrix of all variables is well-conditioned, we normalize each variable (*D*, *D*_*a*_, *M*, *A*, *L*, and *I*) between zero and one by dividing each variable by its largest value over the thirty year interval. The normalization allows us to compare the relative highs and lows of the variables with each other and, as a result, we expect that the trends we find are valid for both the scaled and unscaled data. The consistency of behavior in the raw and normalized data can be seen by comparing rows in Table 2.

The three SEMs consider the state characteristics and outcomes as measured variables, which may be influenced by error terms defined as exogenous latent variables. This formulation is a special case of the general SEM called a simultaneous equation model [31]. In addition to the linear relationships between the measured variables, each model contains a constant intercept term to account for a constant off-set between the variables in the regression analysis [32]. The models are specified as follows. The dependence of *D* on *M* is computed using a SEM with the following straightforward equation defined for each state:

$$D_{i}=\alpha M_{i}+\beta +\gamma \in_{i}$$ (1)

where *D*_*i*_ is the normalized raw number of MVC deaths in year *i*, *M*_*i*_ is the normalized vehicle miles travelled in year *i*, *∈*_*i*_ is a time-varying error, and *α*, *β*, and *γ* are the coefficients computed by the SEM. The dependence of *D*_*a*_ on all the state characteristics, and their interdependences, are computed using a SEM which simultaneously regresses the system of equations defined for each state:

{Dai=α1Mi+α2Ai+α3Li+α4Ii+β1+γ1ϵ1iLi=α5Mi+α6Ai+α7Ii+β2+γ2ϵ2iIi=α8Mi+α9Ai+β3+γ3ϵ3iAi=α10Mi+β4+γ4ϵ4i (2)

where *D*_*ai*_ is the percent MVC deaths involving alcohol in year *i*, *A*_*i*_ is the normalized per capita alcohol consumption in year *i*, *L*_*i*_ and *I*_*i*_ are the proportion of adopted laws and state ideology in year *i*, *∈*_*1i*_,…, *∈*_*4i*_ are time-varying errors, and *α*_1_,…,*α*_*10*_, *β*_*1*_,_…,_ *β*_*4*_, and *γ*_*1*_,…,*γ*_*4*_ are the coefficients computed by the SEM. The dependence of *D/M* on all other state characteristics, and their interdependences, are computed using a SEM with the equation defined for each state:

{(D/M)i=α11Ai+α12Li+α13Ii+β5+γ5ϵ5iLi=α14Ai+α15Ii+β6+γ6ϵ6iIi=α16Ai+β7+γ7ϵ7i (3)

where (*D/M)*_*i*_ is the normalized MVC deaths scaled by the vehicle miles travelled in year *i*, *∈*_*5i*_,…, *∈*_*7i*_ are time-varying errors, and *α*_11_,…,*α*_*16*_, *β*_*5*_,_…,_ *β*_*7*_, and *γ*_*5*_,…,*γ*_*7*_ are the coefficients computed by the SEM. This analysis is performed using the statistics software R (version 3.1.2) [33] with both built-in functions [32] and the associated lavaan package for SEM [33, 34]. The models and statistical significances of the coefficients are reported in Table 1, along with network representations of the models in Fig 3.

Following the analysis in Gallos et al. [23], we define the correlation function as

$$C\left(r\right)\;=\;\frac{1}{\sigma^{2}}\frac{\sum_{ij}{(x}_{i}-x^{*}){(x}_{j}-x^{*})\delta (r_{ij}-r)}{\sum_{ij}\delta (r_{ij}-r)}$$

where *x*_*i*_ is the outcome or characteristic of state *i*, x* is the mean outcome or characteristic over all states, *r*_*ij*_ is the distance between state *i* and state *j*, *σ* ^2^ is the variance $\sum_{i\;=\;1}^{50}{\left(x_{i}-x^{*}\right)}^{2}$, *δ* is the delta symbol taking the value one if its argument is zero and being zero otherwise, and *i*,*j* = 1,…,50. We note that the argument of the *δ* function is equal to zero when the distance between state *i* and state *j* is equal to the target distance set by the argument of the *C* function. As with the correlation coefficient, large magnitude values indicate a strong correlation and values close to zero indicate a weak correlation. However, *C*(*r*) may take values greater than one, since the mean and variance are taken over all states, but the delta function selects only a subset of these as summands. To facilitate comparison of results using both geographic and ideological distances, we normalize the pairwise distances between 0 and 1 by dividing all distances by the largest value in each case. The normalized distances are combined in eleven equal bins, and the largest four bins are merged to lessen the effect of data sparseness at large distances. This analysis is performed with the computing software MATLAB, version 2013b. We note that this correlation analysis is performed on the un-normalized values of each variable.
